# Lean, Non‐Autoimmune Young‐Onset Diabetes in Bangladesh: A Metabolically Obese Phenotype With Disproportionate Insulin Secretory Defect

**DOI:** 10.1002/edm2.70251

**Published:** 2026-06-05

**Authors:** Mashfiqul Hasan, Nusrat Sultana, Kishore Kumar Shil, Sayad Bin Abdus Salam, Koushik Ashraf, Abdullah Al Noman Bhuiyan, Md. Omor Al Masud, Rifat Hossain Ratul, Muhammad Abul Hasanat

**Affiliations:** ^1^ Department of Endocrinology Bangladesh Medical University Dhaka Bangladesh

**Keywords:** Beta‐cell function, insulin resistance, lean diabetes, young‐onset diabetes

## Abstract

**Background:**

Young‐onset diabetes in South Asians frequently manifests in lean individuals, but the metabolic drivers of this phenotype remain poorlycharacterised.

**Objective:**

This study aimed to evaluate the clinical, biochemical, and insulin‐related indices of lean, non‐autoimmune young‐onset diabetes mellitus (DM) among Bangladeshi young adults.

**Methods:**

This comparative cross‐sectional study (2023–2024) enrolled 373 participants (aged 18–34 years) categorised into four groups based on glycemic status and body mass index (BMI): Lean DM (*n* = 53), obese DM (*n* = 125), lean non‐DM (*n* = 78), and obese non‐DM (*n* = 117) from the Young‐diabetes Clinic of the Department of Endocrinology, Bangladesh Medical University, Dhaka after excluding those with positive islet autoantibodies, low C‐peptide, pancreatic pathology, and monogenic variants. β‐cell function (HOMA2‐B) and insulin resistance (HOMA2‐IR) were quantified using the HOMA2 C‐peptide calculator.

**Results:**

Lean DM participants were comparable to other groups in age, sex, and family history, though smoking was more prevalent than in lean controls (*p* < 0.05). Prevalence of central obesity and blood pressure levels of lean DM were similar to those of lean controls, but the lipid profile was comparable to that of obese DM. The median HbA1c was higher in lean DM than in obese DM (11.6% vs. 8.5%; *p* < 0.001), while their median HOMA2‐B was the lowest across all other groups (*p* < 0.01). The HOMA2 − IR in lean DM was lower than in obese DM but significantly higher than in lean controls and mirrored the resistance seen in the obese non‐DM group (*p* < 0.001).

**Conclusion:**

Lean young‐onset diabetes in Bangladesh appears to be a phenotype of disproportionate insulin secretory defect with only modest insulin resistance and metabolic dysfunction. These features highlight the need for targeted screening and individualised management.

## Graphical abstract

Lean young‐onset diabetes in Bangladeshi adults showed marked estimated β‐cell dysfunction, high glycemic burden, modest insulin resistance, and dyslipidemia despite non‐obese BMI. These findings highlight the need for assessment beyond BMI‐based classification and individualised management.

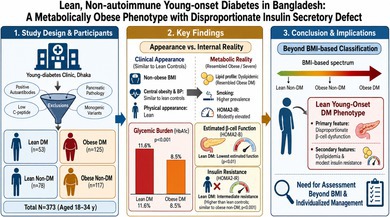

## 1 Introduction

The global rise of young‐onset diabetes is conventionally linked to the increasing prevalence of obesity. Consequently, clinical screening guidelines and public health interventions are largely centred on obesity and insulin resistance. Despite the traditional association with adiposity, individuals with young‐onset diabetes in South Asia frequently lack overt obesity. This lean young‐onset diabetes phenotype is twice as prevalent in South Asians as it is in European cohorts [1]. This “lean diabetes” phenotype is often less emphasised by the healthcare authorities and excluded from standard screening recommendations that prioritise adiposity [2].

The classification of lean, non‐autoimmune diabetes is still controversial. Recently, the concept of Type 5 diabetes has emerged to define this group of individuals, which is thought to be a distinct form of diabetes phenotype [3]. This concept was based on the observation of Lontchi‐Yimagou et al., who evaluated low‐BMI diabetes using a hyperinsulinemic‐euglycemic pancreatic clamp to measure insulin secretory response, endogenous glucose production, and glucose uptake. They observed substantial beta‐cell dysfunction with normal insulin sensitivity and related the condition to undernutrition [4]. This perspective suggests that lean diabetes requires a specialised diagnostic and therapeutic framework.

Conversely, other researchers argue that lean diabetes is not a separate disease but rather one end of a continuous spectrum of type 2 diabetes (T2D) [5]. According to this hypothesis, the phenotype is simply a manifestation of T2D that occurs at a lower BMI threshold and at an earlier age, driven by the unique South Asian phenotype of high visceral adiposity despite low total body weight [6, 7]. While recent large‐scale data from South India suggest a continuum, it also underscores that lean individuals present with a significantly earlier age of onset and more aggressive beta‐cell failure [5].

Despite all these insights, a significant knowledge gap persists, and the issue is still far from clear. Current literature on lean diabetes has predominantly focused on older populations or heterogeneously pooled age groups, leaving the characteristics of this young‐onset phenotype at its onset largely uncharacterised. Given that diabetes in the young is often more aggressive, there is a critical need to shift research focus toward this demographic to elucidate its unique metabolic drivers [8]. Furthermore, there is a paucity of data comparing young lean patients directly to their obese counterparts within the same age range to determine if their metabolic and secretory profiles are truly divergent.

In Bangladesh, a country with a remarkable burden of young‐onset diabetes [9], characterising this phenotype is essential for finding an optimal management approach. It remains unclear whether these young, lean individuals are metabolically healthy except for their glucose levels, or if they harbour the same metabolic obesity (dyslipidemia and resistance) as their obese peers.

This study aimed to evaluate the clinical, biochemical, and metabolic characteristics of lean, non‐autoimmune young‐onset diabetes among Bangladeshi young adults. By comparing beta‐cell function and insulin resistance using the homeostasis model assessment 2 (HOMA2) in lean and obese young adults, both with and without diabetes, we sought to assess whether lean young‐onset diabetes represents a distinct entity or a continuation of the T2D spectrum.

## 2 Methods

### 2.1 Study Design and Participants

This comparative cross‐sectional study recruited young participants from the ‘Young diabetes Clinic’ of the Study on Obesity and Diabetes in Young (SODY) group, Bangladesh Medical University, Dhaka, during January 2023 to December 2024. Young participants with diabetes mellitus (DM) [18–34 years; either newly diagnosed by oral glucose tolerance test (OGTT) or previously known] attending the clinic were recruited consecutively as the DM group. Young attendants and hospital staff who attended the clinic for glycemic screening were enrolled in the Non‐DM group if they had no diabetes (either normal glucose tolerance or prediabetes) on the OGTT. Participants with a history of ketoacidosis, positive autoantibodies [Glutamic Acid Decarboxylase 65 (GAD65), Zinc Transporter 8 (ZnT8), or Anti‐tyrosine phosphatase‐like insulinoma antigen 2/Islet‐Antigen 2 (IA2)], low fasting C‐peptide (< 200 pmol/L), abnormal pancreatic morphology, calcification on imaging, pathogenic or likely pathogenic variants of maturity onset diabetes of young (MODY) or confounding drugs/endocrine diseases impacting glycemic status were excluded.

### 2.2 Sample Size

The sample size was calculated based on the primary objective of comparing β‐cell function (HOMA2‐B) between lean and obese individuals with young‐onset diabetes. Based on previous literature evaluating insulin secretory defects in lean South Asian phenotypes [4], we anticipated a Cohen's d effect size of approximately 0.60 for the difference in HOMA2‐B between the two groups. Utilising the formula for comparison of two independent means [10], with a two‐sided significance level (α) of 0.05% and 80% power (1‐β), a minimum of 44 participants per group was required. We enrolled a larger sample in each group, ensuring the study was sufficiently powered to account for the non‐parametric distribution of HOMA indices.

### 2.3 Data Collection Procedure

Demographic and clinical data were recorded in a case record form by face‐to‐face interview. Anthropometric measurements, including height, weight, waist circumference (WC), hip circumference (HC), and blood pressure, were obtained following standard procedures. Body mass index (BMI), waist‐hip‐ratio (WHR), and waist‐height‐ratio (WHtR) were calculated.

BMI was categorised as underweight (< 18.5 kg/m^2^), healthy weight (18.5–22.9 kg/m^2^), overweight (23–24.9 kg/m^2^), and obesity (≥ 25 kg/m^2^) [11]. For analysis, underweight and healthy weight participants were considered together as ‘lean’, and overweight and obese participants were considered together as ‘obese’. Central obesity was defined as WC ≥ 90 cm in males or ≥ 80 cm in females [12], WHR ≥ 0.9 in males or ≥ 0.85 in females [13], or WHtR ≥ 0.50 [14].

Physical activity was assessed using the Global Physical Activity Questionnaire (GPAQ) version 2. The GPAQ has been evaluated in Bangladeshi adults, showing acceptable test–retest reliability, although criterion validity against objective activity measures was modest; therefore, it is considered suitable for population‐level physical activity assessment rather than precise individual‐level quantification [15, 16]. The metabolic equivalent task (MET) in minutes per week was calculated, and participants were categorised into low (< 600 MET minutes/week), moderate (600–3,000 MET minutes/week), and high (> 3,000 MET minutes/week) physical activity groups.

### 2.4 Assessment of Beta‐Cell Function and Insulin Resistance

Beta‐cell function and insulin resistance were quantified using the HOMA2 computer‐based model (HOMA Calculator, Diabetes Trials Unit, University of Oxford, UK), utilising fasting C‐peptide and fasting glucose concentrations. HOMA2 accounts for the nonlinearity of insulin secretion, making it more accurate for clinical and research purposes [17].

### 2.5 Analytic Methods

Plasma glucose was analysed using the Dimension EXL 200 Integrated Chemistry System (Siemens, Germany) by the glucose‐oxidase method, and HbA1c by High‐Performance Liquid Chromatography. Total cholesterol (TC), triglycerides (TG), and high‐density lipoprotein‐cholesterol (HDL‐C) were measured using an automated analyser (Architect Plus ci8200) by enzymatic methods. Low‐density lipoprotein cholesterol (LDL‐C) was calculated using the Friedewald formula when TG levels allowed calculation; LDL‐C was not calculated in participants with markedly elevated TG, where the formula is considered unreliable [18]. C‐peptide, insulin, GAD‐65 Ab, and IA‐2 antibody were measured by the chemiluminescent immunometric assay using MAGLUMI series chemiluminescence immunometricanalyser, MAGLUMI 2000 plus (Snibe, China), and ZnT8 Ab by ELISA (RSR Ltd., UK). Targeted next‐generation sequencing (NGS) was performed on a panel of 14 known MODY genes in a selected subset of study participants.

### 2.6 Statistical Analysis

Quantitative variables were presented as mean ± SD when normally distributed or as median and interquartile range (IQR) when not normally distributed. Data normality was assessed using the Shapiro–Wilk test. The Kruskal–Wallis test with post hoc analysis and the Bonferroni correction for multiple tests was applied to compare groups. For categorical data, Chi‐square tests with Bonferroni correction were applied for comparisons of proportions (post hoc column proportion tests with Bonferroni correction). A *p*‐value < 0.05 was considered statistically significant. Statistical analysis was performed using IBM SPSS Statistics version 25.0.

### 2.7 Declaration of Generative AI and AI‐Assisted Technologies in the Manuscript Preparation Process

During the preparation of this work, the authors used ChatGPT (OpenAI) and Gemini (Google) to refine the language for clarity and academic tone, assist in formatting the statistical methodology, and conceptualise the layout for the graphical abstract. After using this tool/service, the authors reviewed and edited the content as needed and take full responsibility for the content of the article.

### 2.8 Ethical Aspects

The study was conducted in accordance with the Declaration of Helsinki, and written informed consent was obtained from all the participants. The ethical clearance was obtained from the institutional review board of BMU (BSMMU/2023/6509).

## 3 Results

### 3.1 Demographic and Clinical Characteristics

**FIGURE 1:**
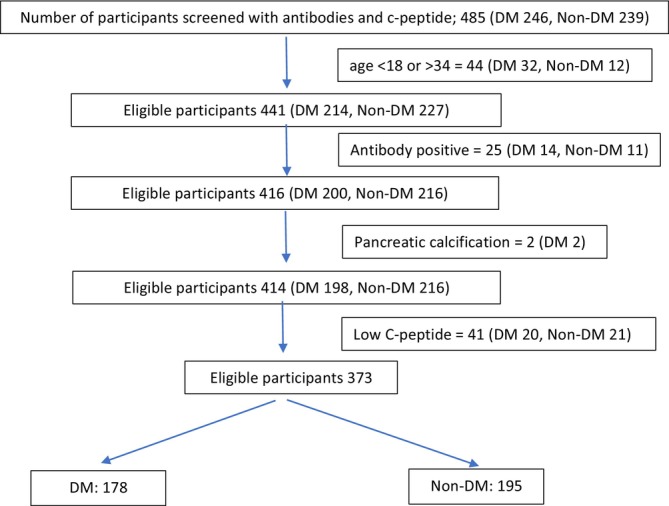
Flowchart for screening and enrollment of study participants (*n* = 373). Ten participants of the DM group had genetic variants of uncertain significance in MODY‐related genes. None of the participants had a pathogenic/likely pathogenic variant.

The study analysed 178 participants with young‐onset diabetes (age 18–34 years), who had negative islet autoantibodies, fasting C‐peptide ≥ 200 pmol/L, and no pancreatic calcification. Ten participants had genetic variants of uncertain significance in MODY‐related genes. As none had a pathogenic/likely pathogenic variant, they were not excluded from the analysis. Among them, 53 were lean DM [9 (5.1%) were underweight, and 44 (24.7%) were of a healthy weight]. On the other hand, 125 were obese DM [41 (23.0%) were overweight, and 84 (47.2%) were obese]. For comparison, the study also enrolled 195 participants in the non‐DM group. Of them, 78 were grouped as lean non‐DM [17 (8.7%) underweight, 61 (31.3%) healthy weight] and 117 as obese non‐DM [46 (23.6%) overweight and 71 (36.4%) obese] (Figure 1).

Age and sex distribution were similar across groups [age: Lean DM 27.0 years (23.5–29.0), lean non‐DM 25.0 years (23.0–28.0), obese non‐DM 29.0 years (26.0–32.0), obese DM 29.0 years (25.0–32.0); female: Lean DM 25/53 (47.2%), lean non‐DM 48/78 (61.5%), obese non‐DM 75/117 (64.1%), obese DM 68/125 (54.4%)]. Family history of DM did not differ significantly between lean DM and the other groups [lean DM 28/53 (52.8%), lean non‐DM 25/78 (32.1%), obese non‐DM 60/117 (51.3%), obese DM 81/125 (64.8%)]. Similarly, low physical activity was similar in all groups [lean DM 32/53 (60.4%), lean non‐DM 45/78 (57.7%), obese non‐DM 75/117 (64.1%), obese DM 34/125 (59.2%)]. Smoking was more common in lean DM than in lean non‐DM [13/53 (24.5%) vs. 5/78 (6.4%), *p* < 0.05].

The lean DM group had a median BMI of 21.1 kg/m^2^ (19.5–22.2), which was similar to lean non‐DM [20.6 kg/m^2^ (18.7–21.9)] but significantly lower than obese non‐DM [25.8 kg/m^2^ (24.0–28.3), *p* < 0.001] and obese DM [26.6 kg/m^2^ (24.4–30.1), *p* < 0.001]. WC, WHR in male and WHtR followed a similar pattern, being comparable between lean DM and lean non‐DM [WC (male) 80.0 cm (76.0–85.0) vs. 78.0 cm (70.5–82.3); WC (female) 79.0 cm (71.8–85.5) vs. 77.5 cm (73.0–84.0); WHR (male) 0.89 (0.83–0.93) vs. 0.85 (0.80–0.89); WHtR 0.49 (0.45–0.53) vs. 0.49 (0.46–0.52)] but lower than obese non‐DM [WC (male) 94.0 cm (87.8–98.3), *p* < 0.001; WC (female) 87.5 cm (83.0–95.0), *p* < 0.001; WHR (male) 0.94 (0.88–0.96), *p* < 0.01; WHtR 0.56 (0.53–0.59), *p* < 0.001] and obese DM [WC (male) 90.0 cm (85.0–98.0), *p* < 0.001; WC (female) 92.0 cm (86.0–101.0), *p* < 0.001; WHR (male) 0.94 (0.90–0.97), *p* < 0.01; WHtR 0.58 (0.55–0.62), *p* < 0.001].

Similarly, the blood pressure of the lean‐DM group [systolic BP 110 mmHg (100–120), diastolic BP 70 mmHg (60–80)] was similar to that of the lean non‐DM [systolic BP 110 mmHg (100–110), diastolic BP 70 mmHg (70–80)], but systolic BP was lower than that of the obese DM [120 mmHg (110–120), *p* < 0.01], and diastolic BP was lower than that of both the obese DM [80 mmHg (70–80), *p* < 0.01] and the obese non‐DM [80 mmHg (70–80), *p* < 0.01]. However, the lean DM group had a frequency of acanthosis nigricans [9/53 (17.0%)] similar to that of obese non‐DM [16/117 (13.7%)], but lower than that of obese DM [65/125 (52.0%), *p* < 0.05] and higher than that of lean non‐DM [1/78 (1.3%), *p* < 0.05] (Table 1).

**TABLE 1:** Demographic and clinical characteristics across the four study groups (*n* = 373).

Variables	Lean DM (*n* = 53)	Lean non‐DM (*n* = 78)	Obese non‐DM (*n* = 117)	Obese DM (*n* = 125)
Age, years	27.0 (23.5–29.0)	25.0 (23.0–28.0)	29.0 (26.0–32.0)	29.0 (25.0–32.0)
Female sex, *n* (%)	25 (47.2)	48 (61.5)	75 (64.1)	68 (54.4)
Family history of DM, *n* (%)	28 (52.8)	25 (32.1)	60 (51.3)	81 (64.8)
Low physical activity, *n* (%)	32 (60.4)	45 (57.7)	75 (64.1)	34 (59.2)
Smoking, *n* (%)	13 (24.5)	5 (6.4)*	11 (9.4)	16 (12.8)
Height, cm	162.0 (151.5–168.0)	156.0 (152.0–166.3)	161.0 (152.5–168.0)	158.0 (152.0–164.0)
Weight, kg	54.2 (47.9–61.0)	50.5 (45.0–55.0)	66.4 (59.8–78.0)***	66.8 (61.0–74.5)***
BMI, kg/m^2^	21.1 (19.5–22.2)	20.6 (18.7–21.9)	25.8 (24.0–28.3)***	26.6 (24.4–30.1)***
Waist circumference in males (cm)	80.0 (76.0–85.0)	78.0 (70.5–82.3)	94.0 (87.8–98.3)***	90.0 (85.0–98.0)***
Waist circumference in females (cm)	79.0 (71.8–85.5)	77.5 (73.0–84.0)	87.5 (83.0–95.0)***	92.0 (86.0–101.0)***
Waist‐hip ratio in male	0.89 (0.83–0.93)	0.85 (0.80–0.89)	0.94 (0.88–0.96)**	0.94 (0.90–0.97)**
Waist‐hip ratio in female	0.91 (0.85–0.96)	0.88 (0.85–0.91)	0.88 (0.84–0.92)	0.91 (0.88–0.97)
Waist‐height ratio	0.49 (0.45–0.53)	0.49 (0.46–0.52)	0.56 (0.53–0.59)***	0.58 (0.55–0.62)***
Systolic BP, mmHg	110 (100–120)	110 (100–110)	110 (103–120)	120 (110–120)**
Diastolic BP, mmHg	70 (60–80)	70 (70–80)	80 (70–80)**	80 (70–80)**
Acanthosis nigricans, *n* (%)	9 (17.0)	1 (1.3)*	16 (13.7)	65 (52.0)*

*Note:* Data are presented as median and IQR, if not mentioned otherwise. Asterisks indicate values that are significantly different in comparison with the Lean DM group. For quantitative variables, *p < 0.05; **p < 0.01; ***p < 0.001, by the independent sample Kruskal–Wallis test with post hoc pairwise comparison and Bonferroni correction for multiple comparisons. For qualitative variables, *p < 0.05 by Chi‐square test with Bonferroni correction for multiple comparisons.

Abbreviations: DM: Diabetes mellitus, BP: Blood pressure.

### 3.2 Biochemical Profile

Median TC of lean DM was 176.5 mg/dL (151.3–200.3), compared with a lower value of 156.5 mg/dL (135.0–183.3) in lean non‐DM (*p* < 0.01), and a similar value of 177.0 mg/dL (153.5–195.0) and 188.0 mg/dL (156.0–215.0) in obese non‐DM and obese DM, respectively. Median LDL‐C was comparable across all groups [lean DM 106.0 mg/dL (82.5–129.0), lean non‐DM 93.4 mg/dL (77.7–112.0), obese non‐DM 104.0 mg/dL (87.7–120.7), obese DM 98.0 mg/dL (78.8–124.0)]. HDL‐C was lower in lean DM [36.5 mg/dL (32.0–46.5)] than in obese non‐DM [45.0 mg/dL (38.0–49.5), *p* < 0.01] and was similar to obese DM [37.0 mg/dL (32.0–43.0), *p* > 0.05]. Median triglyceride was higher in lean DM [177.5 mg/dL (107.5–279.0)] than in lean non‐DM [99.5 mg/dL (67.0–125.0), *p* < 0.001] and obese non‐DM [128.0 mg/dL (91.0–185.5), *p* < 0.05], but was comparable to obese DM [206.0 mg/dL (145.0–350.0), *p* > 0.05].

FPG and 2hPG were statistically similar in the lean [FPG 11.7 mmol/L (7.5–15.8), 2hPG 18.0 mmol/L (12.0–24.0)] and obese DM [FPG 9.3 mmol/L (6.9–13.4), 2 h PG 15.6 mmol/L (12.4–20.4)] groups, with a trend of higher values in the lean DM group. HbA1c was significantly higher in lean DM than in obese DM [11.6% (10.1–12.6) vs. 8.5% (7.0–10.2), *p* < 0.001] (Table 2).

**TABLE 2:** Biochemical profile, insulin secretion, and insulin resistance indices across the four study groups (*n* = 373).

Variables	Lean DM (*n* = 53)	Lean non‐DM (*n* = 78)	Obese non‐DM (*n* = 117)	Obese DM (*n* = 125)
Total cholesterol (mg/dL)	176.5 (151.3–200.3)	156.5 (135.0–183.3)**	177.0 (153.5–195.0)	188.0 (156.0–215.0)
LDL‐C (mg/dL)	106.0 (82.5–129.0)	93.4 (77.7–112.0)	104.0 (87.7–120.7)	98.0 (78.8–124.0)
HDL‐C (mg/dL)	36.5 (32.0–46.5)	42.5 (36.0–49.0)	45.0 (38.0–49.5)**	37.0 (32.0–43.0)
TG (mg/dL)	177.5 (107.5–279.0)	99.5 (67.0–125.0)***	128.0 (91.0–185.5)*	206.0 (145.0–350.0)
FPG (mmol/L)	11.7 (7.5–15.8)	5.0 (4.8–5.4)***	5.1 (4.9–5.6)***	9.3 (6.9–13.4)
2hPG (mmol/L)	18.0 (12.0–24.0)	5.6 (4.8–6.6)***	6.4 (5.6–7.4)***	15.6 (12.4–20.4)
HbA1c (%)	11.6 (10.1–12.6)	—	—	8.5 (7.0–10.2)***
Fasting C‐peptide (pmol/L)	741.4 (529.6–1012.9)	690.1 (512.2–921.0)	993.0 (741.4–1264.4)*	1297.5 (845.7–1845.3)***
Fasting insulin (μIU/mL)	9.0 (5.1–13.6)	8.4 (6.4–11.2)	12.0 (10.1–16.4)**	12.6 (9.4–16.7)**
HOMA2‐B	34.5 (21.8–58.1)	126.8 (96.8–155.4)***	154.2 (125.8–192.2)***	62.7 (40.1–123.6)**
HOMA2‐S	38.8 (26.5–62.6)	66.0 (49.8–87.5)***	45.3 (35.2–60.9)	25.8 (17.8–40.7)**
HOMA2‐IR	2.58 (1.61–3.78)	1.52 (1.14–2.02)***	2.21 (1.64–2.84)	3.88 (2.46–5.64)**

*Note:* Lipid profile was not measured in 1 lean DM and in 2 obese DM. LDL‐C could not be calculated in 3 lean DM, 21 obese DM, and 1 obese non‐DM due to high TG levels. HbA1c available in 34 lean DM (64.2%), 90 Obese DM (72.0%). Fasting Insulin available in 36 lean DM (67.9%), 72 obese DM (57.6%), 77 lean non‐DM (98.7%), 113 obese non‐DM (96.6%). For quantitative variables, *p < 0.05; **p < 0.01; ***p < 0.001, by Independent sample Kruskal–Wallis test with post hoc pairwise comparison and Bonferroni correction for multiple comparisons. For qualitative variables, *p < 0.05 by Chi‐square test with Bonferroni correction for multiple comparisons.

Abbreviations: DM: Diabetes mellitus, BP: Blood pressure.

### 3.3 Beta‐Cell Function and Insulin Resistance Indices

Fasting C‐peptide and fasting insulin levels of lean DM [C‐peptide 741.4 pmol/L (529.6–1012.9), insulin 9.0 μIU/mL (5.1–13.6)] were statistically similar to those of lean non‐DM [C‐peptide 690.1 pmol/L (512.2–921.0), insulin 8.4 μIU/mL (6.4–11.2)], but significantly lower than those of obese DM [C‐peptide 1297.5 pmol/L (845.7–1845.3), *p* < 0.001; insulin 12.6 μIU/mL (9.4–16.7), *p* < 0.01] and obese non‐DM [C‐peptide 993.0 pmol/L (741.4–1264.4), *p* < 0.05; insulin 12.0 μIU/mL (10.1–16.4), *p* < 0.01].

HOMA2‐B was significantly lower in lean DM [34.5 (21.8–58.1)] than in all other groups [lean non‐DM 126.8 (96.8–155.4), *p* < 0.001; obese non‐DM 154.2 (125.8–192.2), *p* < 0.001; obese DM 62.7 (40.1–123.6), *p* < 0.01]. On the other hand, HOMA2‐IR of lean DM [2.58 (1.61–3.78)] was significantly higher than that of lean non‐DM [1.52 (1.14–2.02), *p* < 0.001], similar to that of obese non‐DM [2.21 (1.64–2.84)], but lower than that of obese DM [3.88 (2.46–5.64), *p* < 0.01]. Consistently, median HOMA2‐S was 38.8 (26.5–62.6) in lean DM, compared with 66.0 (49.8–87.5) in lean non‐DM (*p* < 0.001), 45.3 (35.2–60.9) in obese non‐DM (*p* > 0.05), and 25.8 (17.8–40.7) in obese DM (*p* < 0.01) (Table 2).

## 4 Discussion

In this study of young Bangladeshi adults, we evaluated young lean individuals with DM who were negative for islet autoantibodies and had no pancreatic abnormality or secondary cause of diabetes. This lean young‐diabetes group was characterised by lower adiposity and markedly reduced β‐cell secretory capacity. Yet, they showed metabolic dysfunction, with insulin resistance comparable to that of obese non‐DM participants and lipid and glucose profiles resembling those of obese DM participants. These divergent patterns support the notion that lean diabetes is not merely an early or mild stage of obesity‐related T2D and may require a different treatment algorithm from their obese counterparts.

Lean or non‐obese diabetes has attracted researchers' attention for a long time. However, there is an inconsistency in the definition of BMI cut‐offs. Some authors defined lean DM as only low BMI (less than normal) [4, 5, 7, 19], while others included both normal‐ and low‐BMI DM patients as lean DM [20]. We labelled both normal‐ and low‐BMI DM as ‘lean DM’ because of the small number of individuals in the low‐BMI group; nevertheless, the normal‐ and low‐BMI groups showed similar phenotypic and metabolic features in our study participants (Table S1 and Table S2). However, the appropriateness of BMI for categorising participants is debatable, as BMI often fails to reflect an individual's metabolic characteristics [21]. Yet, the present study used it in the context of current practice.

In South Asian populations, visceral adiposity and ectopic fat deposition (in the liver and pancreas) often occur at much lower BMI thresholds than in Western cohorts [22]. This likely explains why the lean DM group exhibited significant acanthosis nigricans and dyslipidemia, despite their lean appearance. Unlike a recent study that found no increased insulin resistance in low‐BMI diabetes, our lean DM group demonstrated clear resistance compared to lean controls [4]. This discrepancy may be due to our inclusion of ‘healthy weight’ (18.5–22.9 kg/m^2^) individuals, who in South Asia may already be at a metabolic disadvantage.

Comparable lean, non‐autoimmune diabetes phenotypes have been reported outside Asia, particularly among Black African adults. In Uganda, Kibirige et al. found that lean adults with newly diagnosed non‐autoimmune diabetes had lower adiposity and fewer metabolic syndrome features than non‐lean participants but showed reduced β‐cell secretory responses [23]. Similarly, another study reported a substantial burden of diabetes among lean Ghanaian adults across rural, urban, and migrant settings, with cardiometabolic and environmental factors contributing to risk [24]. These findings support the concept that lean diabetes is not unique to South Asians. However, both studies included broader adult populations rather than young‐onset diabetes specifically; therefore, comparison with our young‐focused study should be cautious. The balance between β‐cell dysfunction and insulin resistance may vary across populations, age groups, nutritional histories, and environmental contexts.

While insulin resistance was present, the defining characteristic of our young lean DM group was profound beta‐cell dysfunction. The median HOMA2‐B was strikingly low, significantly worse than that of the obese DM group. This suggests a double pathophysiology: Severe defect in insulin secretion coupled with moderate insulin resistance that the failing beta cells cannot overcome. The marked hyperglycemia observed in the lean DM group (median HbA1c 11.6%) may have contributed to impaired β‐cell function through glucotoxicity; however, this possibility cannot be confirmed from the present cross‐sectional data. The high prevalence of smoking in this group also warrants attention, as tobacco use is a known risk factor for oxidative stress and beta‐cell apoptosis [25].

Several plausible mechanisms may explain the coexistence of marked insulin secretory defect with modest insulin resistance in this lean young‐adult population. First, South Asians may develop visceral and ectopic fat deposition at relatively low BMI, leading to insulin resistance despite a non‐obese body habitus. Second, early‐life undernutrition, low birth weight, or adverse intrauterine programming may limit β‐cell reserve, making individuals vulnerable to diabetes when later exposed to calorie‐dense diets, physical inactivity, or weight gain within the normal BMI range. Third, genetic or epigenetic susceptibility affecting β‐cell development, insulin secretion, or fat distribution may contribute to this phenotype. Therefore, the observed phenotype likely reflects a combination of limited β‐cell reserve and metabolically adverse fat distribution rather than a single pathophysiological mechanism.

Lean DM is of particular concern in low‐ and middle‐income countries (LMICs) and is also linked to lower socioeconomic status [20]. Historically, lean diabetes in LMICs was often categorised as malnutrition‐related diabetes mellitus (MRDM). However, the lean DM group in the present study did not show key features traditionally associated with the classic MRDM definition. The presence of dyslipidemia and modest insulin resistance suggests that the observed phenotype is unlikely to be explained by malnutrition alone.

These findings have several clinical implications. Current T2D guidelines usually prioritise insulin sensitisers and weight‐loss‐centric therapies [glucagon‐like peptide‐1 receptor agonists (GLP‐1 RA) and sodium‐glucose cotransporter‐2 inhibitors (SGLT2i)]. However, for a phenotype defined by severe insulin deficiency and high HbA1c, early consideration of insulin therapy or insulin secretagogues may be clinically appropriate in selected patients to achieve rapid glycemic control, although treatment‐response studies are needed. Furthermore, the relatively high prevalence of smoking among lean diabetes patients highlights modifiable risk factors that may exacerbate β‐cell failure and vascular risk.

Our study has several strengths, including the systematic exclusion of autoimmune and secondary causes of diabetes and the recruitment of a young population in a high‐burden region. Although the lean DM manifests at a relatively early age, no previous studies have focused exclusively on young individuals. However, several limitations should be acknowledged. Newly diagnosed cases of DM often have a tendency toward weight loss, which may be responsible for the admixture of obese and lean individuals. LDL‐C was estimated using the Friedewald formula rather than measured directly, and LDL‐C values were unavailable for participants with markedly elevated triglycerides in whom the formula was unreliable. The cross‐sectional design, reliance on surrogate measures of insulin secretion and resistance rather than clamp studies, lack of formal assessment of nutritional status, and the absence of longitudinal follow‐up to assess progression or response to therapy are the other limitations. Future longitudinal studies using gold‐standard clamp techniques are needed to further elucidate the kinetics of insulin secretion in this group.

In conclusion, lean young‐onset diabetes in Bangladesh appears to be a phenotype of disproportionate insulin secretory defect with only modest insulin resistance and metabolic dysfunction. These features highlight the need for targeted screening and individualised management. Further mechanistic studies are warranted to clarify its aetiology and natural history.

## Author Contributions

**Koushik Ashraf:** conceptualization, data curation, formal analysis, writing – review and editing. **Sayad Bin Abdus Salam:** conceptualization, data curation, formal analysis, writing – review and editing. **Abdullah Al Noman Bhuiyan:** conceptualization, data curation, formal analysis, writing – review and editing. **Muhammad Abul Hasanat:** conceptualization, methodology, supervision, project administration, funding acquisition, writing – review and editing. **Nusrat Sultana:** conceptualization, methodology, validation, writing – review and editing, project administration, supervision. **Rifat Hossain Ratul:** conceptualization, data curation, formal analysis, writing – review and editing. **Mashfiqul Hasan:** conceptualization, methodology, writing – review and editing, writing – original draft, visualization, formal analysis, data curation, investigation. **Md. Omor Al Masud:** conceptualization, data curation, formal analysis, writing – review and editing. **Kishore Kumar Shil:** conceptualization, data curation, formal analysis, investigation, writing – review and editing.

## Funding

This work was supported by Bangladesh Medical University under grant number BSMMU/2023/13314(6).

## Disclosure

The authors have nothing to report.

## Conflicts of Interest

The authors declare no conflicts of interest.

## Supporting information


**Table S1:** Comparison of demographic and clinical characteristics of lean DM participants (*n* = 53).
**Table S2:** Comparison of metabolic profile and insulin indices of lean DM participants (*n* = 53).

## Data Availability

The data that support the findings of this study are available from the corresponding author upon reasonable request.
