# Supplementary material for: Lean, Non‐Autoimmune Young‐Onset Diabetes in Bangladesh: A Metabolically Obese Phenotype With Disproportionate Insulin Secretory Defect
Source: Endocrinol Diabetes Metab. 2026 Jun 5;9(4):e70251. doi: 10.1002/edm2.70251 (PMC13238763; doi:10.1002/edm2.70251)
Supplement: Supplementary file 1 — Table S1: Comparison of demographic and clinical characteristics of lean DM participants (n = 53). Table S2: Comparison of metabolic profile and insulin indices of lean DM participants (n = 53). [file EDM2-9-e70251-s001.docx]

**Supplementary Table 1**. Comparison of demographic and clinical characteristics of lean DM participants (n = 53)

| **Variables** | **Low-BMI DM (n=9)** | **Normal BMI DM (n=44)** | ***p-value** |
| --- | --- | --- | --- |
| Age, years | 27.0 (23.5-29.0) | 27.0 (24.0-29.0) | 0.201 |
| Female sex, n (%) | 4 (44.4) | 21 (47.7) | 1.000 |
| Family history of DM, n % | 5 (55.6) | 23 (52.3) | 1.000 |
| Low physical activity, n % | 6 (66.7) | 26 (59.1) | 1.000 |
| Smoking, n % | 2 (22.2) | 11 (25.0) | 1.000 |
| BMI, kg/m² | 18.0 (16.2-18.1) | 21.7 (20.6-22.3) | **0.004** |
| Waist circumference in males (cm) | 71.0 (67.5-71.5) | 80.5 (78.0-85.0) | 0.053 |
| Waist circumference in females (cm) | 64.0 (44.3-71.8) | 81.0 (74.0-86.0) | 0.096 |
| Waist-hip ratio in male | 0.82 (0.79-0.82) | 0.90 (0.86-0.93) | 0.326 |
| Waist-hip ratio in female | 0.82 (0.59-0.86) | 0.92 (0.86-0.97) | 0.096 |
| Waist-height ratio | 0.42 (0.39-0.42) | 0.50 (0.47-0.54) | **0.033** |
| Systolic BP, mmHg | 100 (90-105) | 110 (100-120) | 0.122 |
| Diastolic BP, mmHg | 70 (60-70) | 70 (63-80) | 0.122 |
| Acanthosis nigricans, n% | 1 (11.1) | 8 (18.2) | 1.00 |

Data are presented as median and IQR, if not mentioned otherwise

*For quantitative variables, by the independent sample median test

For qualitative variables, by Fisher’s exact test

DM: diabetes mellitus, BP: blood pressure

**Supplementary Table 2**. Comparison of metabolic profile and insulin indices of lean DM participants ­(n=53)

| **Variables** | **Low-BMI DM (n=9)** | **Normal BMI DM (n=44)** | **p-value** |
| --- | --- | --- | --- |
| Total cholesterol (mg/dL) | 173.0 (159.5-183.8) | 181.0 (149.0-212.0) | 0.463 |
| LDL-C (mg/dL) | 106.3 (77.0-122.0) | 106.0 (82.5-133.0) | 0.746 |
| HDL-C (mg/dL) | 34.5 (32.3-52.5) | 38.0 (32.5-46.0) | 0.463 |
| TG (mg/dL) | 94.5 (77.8-294.3) | 178.0 (130.0-239.0) | 0.463 |
| FPG (mmol/L) | 22.0 (18.6-28.7) | 13.0 (10.9-16.3) | 0.850 |
| 2hPG (mmol/L) | 27.3 (26.6-33.0) | 20.5 (16.9-23.9) | 0.950 |
| HbA1c (%) | 14.3 (10.9-17.2) | 11.4 (9.8-12.4) | 0.595 |
| Fasting C-peptide (pmol/L) | 516.4 (278.0-711.7) | 723.2 (490.7-1020.3) | 0.161 |
| Fasting insulin (μIU/mL) | 7.8 (3.1-13.7) | 9.0 (5.8-14.0) | 0.655 |
| HOMA2-B | 34.6 (11.0-80.0) | 34.4 (23.2-59.1) | 0.950 |
| HOMA2-S | 50.1 (10.2-115.7) | 38.6 (27.4-57.7) | 0.852 |
| HOMA2-IR | 2.00 (0.89-44.3) | 2.60 (1.74-3.65) | 0.950 |

Data are presented as median and IQR,

*For quantitative variables, by the independent sample median test

For qualitative variables, by Fisher’s exact test

DM: diabetes mellitus, BP: blood pressure
